# Activated microglia desialylate their surface, stimulating complement receptor 3‐mediated phagocytosis of neurons

**DOI:** 10.1002/glia.23757

**Published:** 2019-11-27

**Authors:** David H. Allendorf, Mar Puigdellívol, Guy C. Brown

**Affiliations:** ^1^ Department of Biochemistry University of Cambridge Cambridge UK

**Keywords:** complement receptor 3, desialylation, inflammation, microglia, neuraminidase, neurodegeneration, phagocytosis

## Abstract

The glycoproteins and glycolipids of the cell surface have sugar chains that normally terminate in a sialic acid residue, but inflammatory activation of myeloid cells can cause sialidase enzymes to remove these residues, resulting in desialylation and altered activity of surface receptors, such as the phagocytic complement receptor 3 (CR3). We found that activation of microglia with lipopolysaccharide (LPS), fibrillar amyloid beta (Aβ), Tau or phorbol myristate acetate resulted in increased surface sialidase activity and desialylation of the microglial surface. Desialylation of microglia by adding sialidase, stimulated microglial phagocytosis of beads, but this was prevented by siRNA knockdown of CD11b or a blocking antibody to CD11b (a component of CR3). Desialylation of microglia by a sialyl‐transferase inhibitor (3FAx‐peracetyl‐Neu5Ac) also stimulated microglial phagocytosis of beads. Desialylation of primary glial‐neuronal co‐cultures by adding sialidase or the sialyl‐transferase inhibitor resulted in neuronal loss that was prevented by inhibiting phagocytosis with cytochalasin D or the blocking antibody to CD11b. Adding desialylated microglia to glial‐neuronal cultures, in the absence of neuronal desialylation, also caused neuronal loss prevented by CD11b blocking antibody. Adding LPS or Aβ to primary glial‐neuronal co‐cultures caused neuronal loss, and this was prevented by inhibiting endogenous sialidase activity with N‐acetyl‐2,3‐dehydro‐2‐deoxyneuraminic acid or blockage of CD11b. Thus, activated microglia release a sialidase activity that desialylates the cell surface, stimulating CR3‐mediated phagocytosis of neurons, making extracellular sialidase and CR3 potential treatment targets to prevent inflammatory loss of neurons.

## Main points

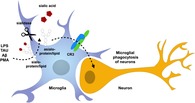

- Inflammatory activated microglia increase surface desialylation.
- Desialylation of microglia leads to increased phagocytic activity via integrin complement receptor 3.
- Blockage of sialidase reduced neuronal loss induced by inflammation.

## 1 INTRODUCTION

Microglia are macrophages resident in the central nervous system (CNS), and are the main mediators of inflammation and phagocytosis in the CNS. They are of myeloid origin and therefore related to phagocytes in the periphery (Saijo & Glass, 2011). Microglia defend the brain against invading pathogens, but also engulf and remove apoptotic neurons, necrotic neurons, debris, protein aggregates, synapses and axons (Rabinowicz & Courten‐Myers, 1996; Vilalta & Brown, 2018). Microglia phagocytose less‐active synapses during development to shape neuronal networks, and too little of this “synaptic pruning” may contribute to autism, and too much pruning may contribute to schizophrenia (Neniskyte & Gross, 2017; Sekar et al., 2016). During neurodegenerative diseases, such as Alzheimer's disease, excessive microglial phagocytosis of synapses and live neurons may cause the neurodegeneration (Vilalta & Brown, 2018). Thus, it is important to understand how microglial phagocytosis is regulated.

Microglial phagocytosis can be activated by exposure to inflammatory stimuli such as bacterial lipopolysaccharide (LPS). LPS stimulates microglia to express and release complement components C1q and C3, which can opsonize by binding amyloid, synapses or neurons and encouraging microglia to phagocytose such tagged targets via the microglial complement receptor 3 (CR3). CR3 is an integrin receptor, consisting of CD11b and CD18, detecting iC3b‐opsonized targets, and is a phagocytic receptor mediating microglial phagocytosis of synapses and dendrites (Linnnartz, Kopatz, Tenner, & Neumann, 2011; Schafer et al., 2012). CR3 can be regulated by “inside‐out” activation via binding of cytoplasmic proteins inducing a conformational change in the extracellular ligand binding site (Takagi, Petre, Walz, & Springer, 2002). However, activation of neutrophils results in desialylation of CR3, which stimulates its ability to bind to ligands (Feng et al., 2011). This suggests that CR3 can be activated “outside‐in” by desialylation, or more generally that in inflammatory conditions phagocytic receptors may be “licensed” to operate by desialylation (Feng et al., 2011).

Sialic acids are a family of structurally‐related and negatively‐charged monosaccharides, which typically terminate the N‐glycans of membrane‐bound proteins or glycolipids when linked to the penultimate galactose residue via α2,3‐ or α2,6‐linkage. Sialic acids constitute the main negative charge of the cell surface, and regulate cell attachment and interactions via multiple mechanisms (Schauer, 1985; Varki, 1997). The removal of these terminal sialic acid residues is termed “desialylation” and, in mammals, can be mediated by four different sialidase/neuraminidase isoenzymes (Neu1‐Neu4). Neu1 has been reported on the surface of mature macrophages (Liang et al., 2006) and activated neutrophils (Feng et al., 2011), and this activity can increase phagocytic signaling (Seyrantepe et al., 2010).

Desialylation of neurons promotes binding of C1q and C3b, which in turn encourages microglia to phagocytose dendrites via CR3 (Linnnartz et al., 2011). Mutations of sialyl‐transferase genes result in desialylation and multiple diseases of brain development and function in humans, and knockout of homologous genes in mice results in extensive loss of myelin, dendrites and neurons (Yoo et al., 2015). This was suggested to be as a result of desialylation of neurons and myelin, but could equally be due to changes in sialylation of microglia. We recently found that LPS stimulated BV‐2 microglia to release a sialidase activity that desialylates co‐cultured cells (Nomura, Vilalta, Allendorf, Hornik, & Brown, 2017). However, the functions and implications of this activity on the microglial cell surface are unknown. More generally, it is not known whether sialylation of the microglial surface changes in inflammatory conditions, and whether changes in microglial sialylation affect microglial function.

Here we demonstrate that primary microglial cells undergo desialylation after exposure to a variety of stimuli: LPS, fibrillar amyloid beta (Aβ), phorbol 12‐myristate 13‐acetate (PMA) and rTAU protein. Importantly, removal of sialic acid residues on microglial cells enhances their ability to phagocytose and this was blocked by knockdown of CD11b. In neuronal‐glial co‐cultures desialylation induces neuronal loss that is inhibited by a blocking antibody against CD11b. Moreover, the sialidase inhibitor N‐acetyl‐2,3‐dehydro‐2‐deoxyneuraminic acid (DANA) blocked neuronal loss in LPS‐ or Aβ‐stimulated neuronal‐glial cultures. These results indicate novel ways of blocking microglia phagocytosis and inflammatory neuronal loss.

## 2 MATERIALS AND METHODS

### 2.1 Materials

All cell culture reagents were from Invitrogen (Paisley, UK). All chemicals were purchased from Sigma‐Aldrich (St. Louis, MO) unless indicated otherwise. Neuraminidase from *Clostridium perfringens* was from Sigma‐Aldrich. 3‐FAx‐peracetyl‐Neu5Ac was from Merck Millipore (Burlington, MA). Carboxylated Nile red 5 μm beads were from Spherotech (Chicago, IL). CD11b‐targeting, nontargeting siRNA, Lipofectamine 3000 reagent and mouse IgG2a isotype control were purchased from Thermo Scientific (Waltham, MA). Mouse monoclonal anti‐CD11b (clone OX‐42) antibody was from Serotec AbD (Hercules, CA). Recombinant human Tau protein (isoform 2N4R) was a kind gift from Dr Vilmante Borutaite. FITC‐labeled human fibrinogen was from Molecular Innovations (Novi, MI).

### 2.2 Cell culture and treatments

All experiments were performed in accordance with the U.K. Animals (Scientific Procedures) Act (1986) and approved by the Cambridge University local ethical committee. The immortalized microglial cell line BV‐2 were maintained as previously described (Blasi, Barluzzi, Bocchini, Mazzolla, & Bistoni, 1990). Glial cultures were prepared from postnatal Days 5–7 rat cortex. Mixed neuronal‐glial co‐cultures were from 5 to 7 day old rat or mouse cerebella. The extraction and cultivation of both these cultures are described elsewhere (Neher et al., 2011). LPS from *Salmonella enterica* serotype typhimurium was added at 100 ng/mL for 24 or 72 hr where indicated. Aβ 1–42 (Anaspec (Fremont, CA)) was prepared as previously described and fibrilized over 24 hr at 37°C. Fibrillar Aβ was used at 3 μM for 24 hr or 1 μM for 72 hr as indicated. PMA was used at 1 μM final concentration. 2N4R Tau protein was used at 1.5 μM over 24 hr. Exo‐sialidase from *C. perfringens* was added at 200 mU/mL for 1 hr prior to the phagocytosis assays. For long term treatments in mixed neuronal‐glia cultures sialidase was added at 80 mU/mL. Desialylation by the sialyl‐transferase inhibitor 3‐FAx‐peracetyl‐Neu5Ac was achieved by adding the inhibitor 1 or 2 days to primary or BV‐2 cultures, respectively (both at 100 μM).

Neuronal‐glial co‐cultures were treated with the sialyl‐transferase inhibitor at 80 μM for 3 days. DMSO was used a vehicle in these experiments. CD11b blocking or isotype control antibodies were added at 5 μg/mL in bead‐uptake experiments 1 hr prior to desialylation. In mixed neuronal‐glial experiments CD11b or isotype control and cytochalasin D (3 μg/mL and 1 μM final concentration, respectively) were added at 2 day of the treatments. For neuronal‐glial experiments with added microglia, microglia were either desialylated by pretreatment with 100 μM sialyl‐transferase inhibitor for 1 day in co‐culture with astrocytes, or by pretreatment with *C. perfringens* sialidase at 200 mU/mL for 1 hr. Desialylated microglia were added at 60,000 cells per treated well in presence of CD11b or isotype control antibody. Neuronal loss was evaluated 24 hr post‐treatment. Sialidase inhibitor DANA was added for 3 days to mixed neuronal‐glial cultures at 400 μM.

### 2.3 Neuraminidase activity assay

Endogenous neuraminidase activity on BV‐2 or primary microglia was assessed by an Amplex Red Neuraminidase Assay Kit (Life Technologies, Carlsbad, CA) following the manufacturer's instructions. Briefly, 1 × 10^5^ cells were cultured in phenol red‐free DMEM and treated accordingly. Twenty‐four hours post‐treatment cells were washed with warm phenol red‐free DMEM and then subjected to a reagent mix containing 50 μM Amplex Red reagent, 0.1 unit/mL HRP, 2 unit/mL galactose oxidase (from *Dactylium dendroides*) and 250 μg/mL fetuin (from fetal calf serum) in reaction buffer containing 50 mM Tris–HCl (pH 7.2) and 1 mM CaCl2 for 30 minutes at 37°C. Fluorescence was measured on an Optima Plate Reader (BMG Technologies, Aylesbury, UK) with 530 nm excitation and 590 nm emission detection.

### 2.4 Desialylation staining and fibrinogen binding

Desialylation was also detected by staining cells with FITC‐coupled peanut agglutinin (PNA‐FITC). Briefly, detached cells were incubated at 5 μg/mL PNA‐FITC in PBS for 30 min at room temperature, washed in PBS and then taken to flow cytometry. Similarly, binding of human FITC‐labeled fibrinogen to microglia was assessed as follows. Cells were incubated with 100 μg/mL labeled fibrinogen in PBS for 30 min at room temperature and washed in PBS. Binding was assessed by flow cytometry by measuring the mean FL1 intensity of at least 5,000 events.

### 2.5 siRNA‐mediated knockdown

BV‐2 cells at 70–80% confluency were subjected to a lipid:siRNA mix containing 3% (v/v) Lipofectamine 3000 (Thermo Fischer, Waltham, MA) and 60 pmol of either CD11b targeting or scrambled siRNA (Silencer Select siRNA, Thermo Fischer) in serum‐free OptiMEM. Transfection medium was removed after 3 hr incubation at 37°C and replaced by DMEM containing 10% FBS. This procedure was repeated 24 hr after the first transfection. Twenty‐four hours after the second transfection BV‐2 cells were re‐seeded at appropriate density in low‐serum DMEM. At this point RNA was extracted (see below). Phagocytosis assays were typically performed on the following day.

### 2.6 Bead phagocytosis assays

Fluorescent 5 μm beads were added at 0.005% (w/v) to cells and left for 3 or 1 hr to BV‐2 or primary cells, respectively. Media was aspirated and cells washed several times with cold PBS. Uptake of beads into cells was assessed by flow cytometry (Accuri C6 BD) or by imaging with an epifluorescent microscope (Leica DM16000 CS). For cytometry, cells were lifted by trypsinization and re‐suspended in PBS. At least 5,000 cells were analyzed for each treatment replicate. Bead‐uptake could be observed in FL‐3 due to the coupling of the beads to the Nile red dye. For each experiment the percentage of cells containing beads was calculated and normalized to the vehicle‐treated condition. For microscopy, cells were stained for Hoechst 33342 (5 μg/mL) and four microscopic fields per well with at least two wells were quantified. Uptake was calculated by counting beads within cells over total numbers of cells.

### 2.7 RNA isolation and RT‐qPCR

Whole RNA was isolated 24 hr postsecond siRNA treatment using Monarch Total RNA Miniprep Kit (NEB, Ipswich, MA). Using the SuperScript II Reverse Transcriptase Kit (Invitrogen) 1 μg of total RNA was converted into cDNA using random hexamer primers. qPCR was carried out with the Platinum SYBR Green qPCR SuperMix (Invitrogen). Primer pair sequences for CD11b were fwd 5′ATGGACGCTGATGGCAATACC and rev 5′TCCCCATTCACGTCTCCCA. Samples were amplified and measured in a Rotor‐Gene Q cycler (Qiagen, Hilden, Germany). Data was analyzed with the Rotor‐Gene Q software. Comparative concentrations were normalized by the housekeeping gene β‐actin to correct for different efficiencies of reverse transcription.

### 2.8 Cell density measurements

Cell densities were quantified in cultures at 3 day post‐treatment. Cultures were subjected to nuclear stain Hoechst 33342 (5 μg/mL) and microglial stain Alexa 488‐tagged Isolectin GS‐IB4 from *Griffonia simplicifolia* (1 μg/mL). Healthy and apoptotic (chromatin‐condensed) neurons were recognized by their distinct nuclear morphology. Per well four microscopic fields were quantified for a single experiment with *n* = 2 wells per condition.

### 2.9 Statistical analysis

Analysis of data was performed using Graphpad Prism (version 6.0) and data shown represented as a mean of at least *n* = 3 independent experiments ± *SEM*. Statistical significance was assessed by ANOVA followed by Tukey's or Sidak's post hoc test or by *t* tests where indicated. *p*‐Values of *p* ≤ .05 are considered significant.

## 3 RESULTS

### 3.1 Activation of microglia in vitro results in desialylation of microglia and sialidase activity on the surface of primary rat microglia

**Figure 1:**
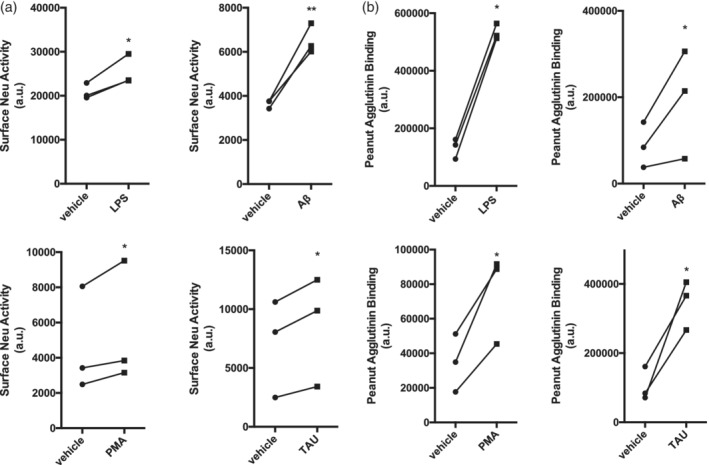
Activation of primary rat microglia stimulates cell surface sialidase activity and results in surface desialylation. (a) Surface neuraminidase (Neu) activity was measured on live rat primary microglia after 24 hr of exposure to fibrillar amyloid beta (Aβ, 3 μM), phorbol myristate acetate (PMA, 1 μM), lipopolysaccharide (LPS, 100 ng/mL) or Tau protein (2N4R isoform, 1.5 μM). Data presented as mean fluorescence signal from individual experiments (*n* = 3). Statistical analysis was performed by paired *t* tests. *p < .05, **p < .01. (b) Binding of the FITC‐conjugated lectin peanut agglutinin (PNA) to primary rat microglia as measured by flow cytometry in the FL‐1 channel. Data presented as mean fluorescence intensities from *n* = 3 individual experiments. Statistical analysis was performed by paired *t* tests, *p < .05

We previously found that microglial BV‐2 cells have increased sialidase activity on the cell surface when stimulated with bacterial LPS (Nomura et al., 2017), so we tested here whether primary rat microglia have increased sialidase activity when activated by a variety of inflammatory stimuli. We found that primary rat microglia incubated with LPS, PMA, fibrillar Aβ and TAU protein have increased sialidase activity on the microglial surface (Figure 1a). Sialidase activity can cause desialylation, so we tested whether LPS, PMA, fibrillar Aβ and TAU would promote desialylation of the microglial cell surface. Desialylation was measured by the binding of peanut agglutinin (PNA) to the cells, as PNA binds galactose residues and this binding is blocked by sialylation. Indeed, we found that all these inflammatory stimuli induced an increase in binding of PNA to the microglia (Figure 1b), indicating desialylation of the cell surface.

### 3.2 Direct desialylation of microglia by a sialidase or sialyl‐transferase inhibitor increases phagocytosis of 5 μm beads

**Figure 2:**
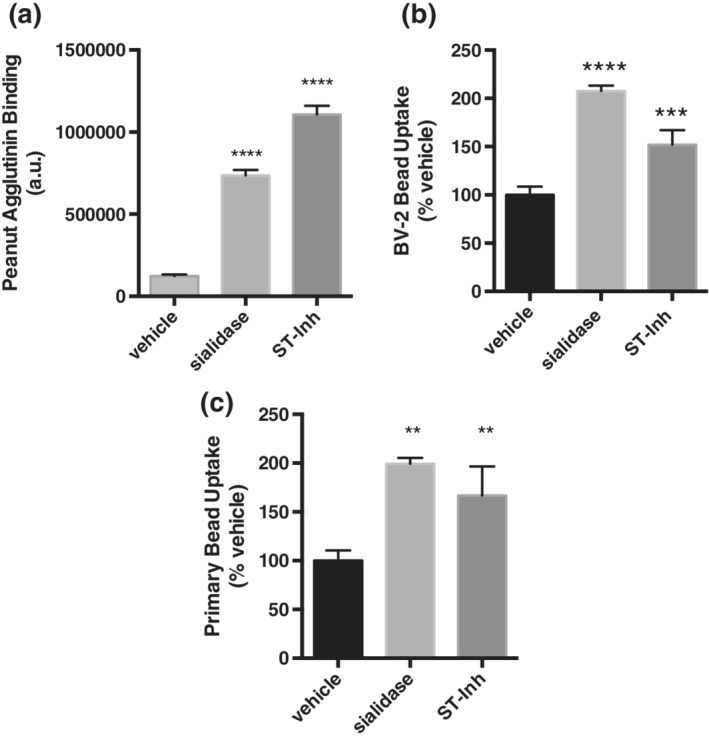
Direct desialylation of microglia by a sialidase or sialyl‐transferase inhibitor increases phagocytosis of 5 μm beads. (a) FITC‐conjugated lectin peanut agglutinin (PNA‐FITC) binding to sialidase treated or sialyl‐transferase inhibitor (ST‐Inh)‐treated BV‐2 microglia. Data presented as mean FL‐1 fluorescence values, with error bars presenting *SEM* of at three independent experiments. Statistical analysis was performed by one‐way ANOVA with Tukey's post hoc test, ****p < .0001. (b) Phagocytic uptake of 5 μm beads by BV‐2 microglia. (c) Phagocytic uptake of 5 μm beads by primary rat microglia. Data presents the percentage of cells containing beads normalized to the vehicle condition. Error bars represent *SEM* of three independent experiments. Statistical analysis was performed by one‐way ANOVA with Sidak's post hoc test **p < .01, ***p < .001, ****p < .0001

To investigate the impact of sialylation on microglial phagocytosis, we used the sialidase enzyme from *C. perfringens* or an inhibitor of sialyl‐transferase enzymes, 3FAx‐peracetyl‐Neu5Ac (ST‐Inh), to effectively desialylate BV‐2 or primary microglia. One hour treatment of BV‐2 microglia with the sialidase enzyme or 1–2 day incubation with the sialyl‐transferase inhibitor led to strong binding of PNA to the microglia (sixfold increase ±0.3 *SEM* for sialidase, ninefold increase ±0.4 for ST‐Inh vs. vehicle), indicating a large decrease in sialylation (Figure 2a). To determine whether desialylation affected the phagocytic behavior of microglial cells, we incubated the cells with 5 μm beads (fluorescent and carboxylated). We observed an increase in the phagocytosis of beads by desialylated BV‐2 (sialidase: 2.1 ± 0.1‐fold increase, ST‐Inh: 1.5 ± 0.1‐fold increase) and primary microglia (twofold increase ±0.1 *SEM* for sialidase, 1.7 ± 0.2‐fold increase for ST‐Inh) compared to vehicle‐treated conditions (Figure 2b,c). Thus, desialylation of microglia, whether induced by stimulating desialylation or inhibiting sialylation, induces an increase in microglial phagocytosis.

### 3.3 Knockdown or blockage of CD11b reduces sialidase‐induced phagocytosis, and desialylation of microglia increases binding of CR3 ligand fibrinogen

**Figure 3:**
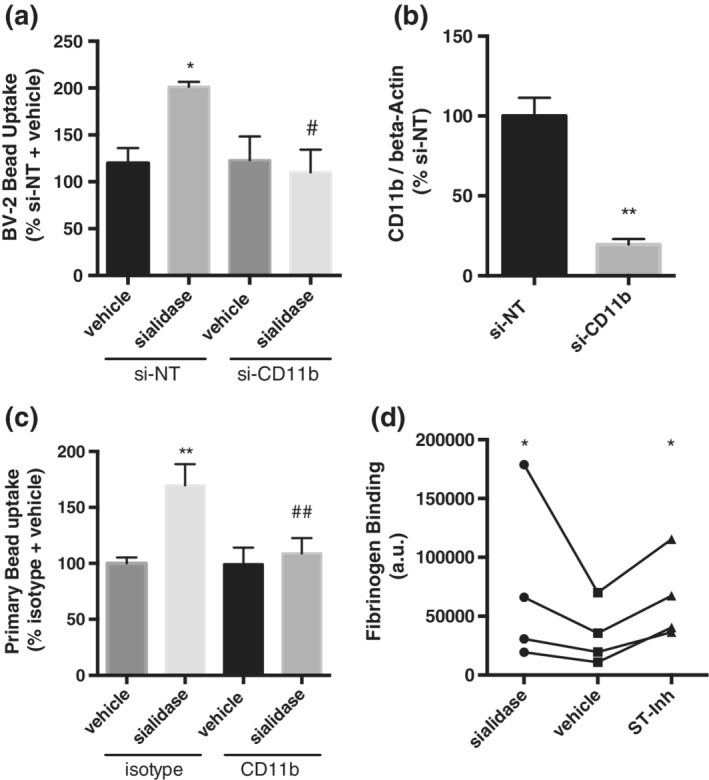
Knockdown or blockage of CD11b reduces sialidase‐induced phagocytosis in BV‐2 cells and primary rat microglia, respectively. (a) Uptake of 5 μm bead into BV‐2 microglia that were pretreated with nontargeting (NT) or CD11b targeting siRNA. Phagocytic uptake was measured in three independent knockdown experiments and data normalized to nontargeting siRNA (si‐NT). Error bars represent *SEM*. Statistical analysis was performed by one‐way ANOVA with Tukey's post hoc test, *p < .5 versus si‐NT control, ^#^ *p* < .5 versus si‐NT sialidase. (b) CD11b expression 48 hr after siRNA mediated knockdown of CD11b. Data was normalized by beta‐actin gene expression and measurements represent three independent knockdown experiments with error bars representing *SEM*. Statistical analysis was performed with an unpaired *t* test, **p < .01. (c) Uptake of beads in primary microglia after stimulation with sialidase in the presence or absence of blocking antibody CD11b or isotype control. *N* = 3 independent phagocytosis assays with error bars representing *SEM*. Statistical analysis was performed by one‐way ANOVA with Tukey's post hoc test, **p < .01 versus isotype + control treatment, ^##^ *p* < .01 versus sialidase + anti‐CD11b treatment. (d) Binding of FITC‐labeled human fibrinogen to vehicle treated or desialylated primary rat microglia. Data presented as mean fluorescence values of three independent binding assays. Statistical analysis was performed by repeat measure (RM) one‐way ANOVA followed by Sidak's post hoc test, *p < .05

CR3 is a key phagocytic receptor of microglia, composed of CD18 and CD11b, which is known to be regulated by sialylation in neutrophils (Feng et al., 2011). So, we tested whether CD11b was involved in the sialidase‐induced increase in phagocytosis. Using a siRNA‐mediated knockdown approach we achieved efficient ablation of CD11b mRNA in BV‐2 microglia (Figure 3b). Interestingly, upon depletion of CD11b, we did not observe a sialidase‐induced increase in phagocytosis in BV‐2 cells as compared to a nontargeting siRNA control (Figure 3a). Similarly, when blocking CD11b with a blocking antibody, we entirely prevented the sialidase‐induced increase in bead phagocytosis by primary rat microglia. (Figure 3c). Thus, CR3 appears to be responsible for the increase in phagocytosis induced by desialylation of microglia. To further confirm the involvement of CR3 in this pathway we tested the binding of a classic CR3 ligand, fibrinogen (Wright et al., 1988), to microglia. We found that desialylated microglia bound more FITC‐labeled human fibrinogen than vehicle‐treated microglia (Figure 3d).

### 3.4 Direct desialylation of glial‐neuronal co‐cultures by sialidase or sialyl‐transferase inhibitor results in neuronal loss, prevented by blocking CD11b or phagocytosis

**Figure 4:**
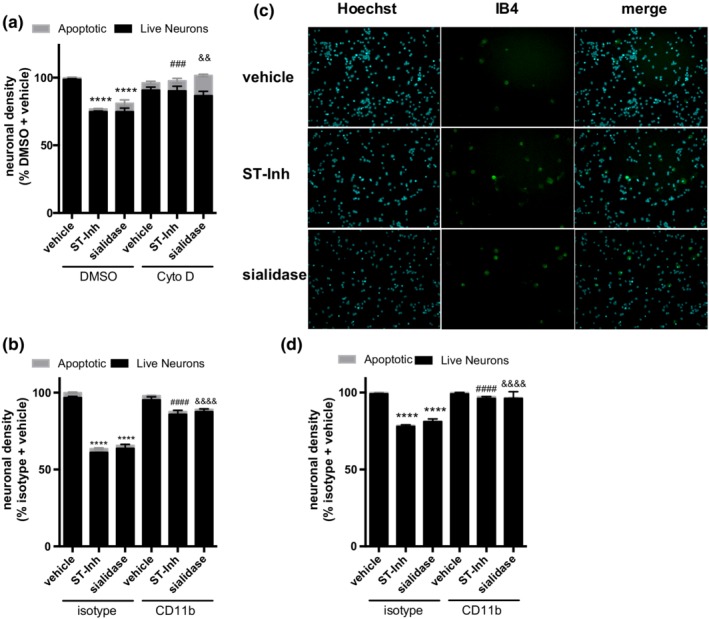
Desialylation causes neuronal loss in glial‐neuronal co‐cultures, which is prevented by blockage of phagocytosis or CD11b. (a and b) Neuronal densities of a mouse (a) and rat (b) neuronal‐glial co‐cultures with indicated treatments. Neurons were counted 3 days after application of desialylating agents ± cytochalasin D (Cyto D) (a) or antibodies (b) added at Day 2 of the treatment. Gray bars represent the percentage of apoptotic neuronal nuclei. Mean neuronal densities of three independent cultures were quantified and normalized to vehicle condition. Error bars represent *SEM*. Statistical analysis was performed by two‐way ANOVA with Sidak's post hoc test. ****p < .0001 versus vehicle treatment, ^###^ *p* < .001 versus sialyl‐transferase inhibitor (ST‐Inh) treatment, ^&&^ *p* < .01 versus sialidase treatment. (c) Representative images of glial‐neuronal co‐cultures imaged 3 days after vehicle, sialidase or ST‐Inh treatment. Cells were stained with nuclei‐staining dye Hoechst 33342 (cyan) and microglia‐staining dye Isolectin‐B4‐Alexa 488 (IB4, green) and visualized at ×20 magnification. (d) Neuronal densities of neuronal‐glial co‐cultures that were treated with desialylated (either by ST‐Inh or sialidase) primary rat microglia. Approximately 60,000 microglia were added to each well ± CD11b neutralizing antibody or isotype control, and neuronal density assessed 1 day later. Data presented as mean of three independent experiments ± SEM. Statistical analysis was performed by two‐way ANOVA with Sidak's post hoc test. ****p < .0001 versus isotype + vehicle, ^####^ *p* < .0001 versus isotype + ST‐Inh, ^&&&&^ *p* < .0001 versus isotype + sialidase [Color figure can be viewed at http://wileyonlinelibrary.com]

Because desialylation of microglia stimulates phagocytosis, we wondered whether it would stimulate microglial phagocytosis of neurons. To assess the effect of desialylation on phagocytosis of live neurons, we determined the effect of adding a sialidase or sialyl‐transferase inhibitor to glial‐neuronal co‐cultures derived from rat or mouse cerebella. As shown in Figure 4a, when desialylated with either sialyl‐transferase inhibitor or sialidase we observed a significant reduction in neuronal density after 3 days (from 100 to 75 ± 1% healthy neurons for sialyl‐transferase inhibitor, and to 75 ± 5% healthy neurons for sialidase). This neuronal loss was prevented by adding an inhibitor of phagocytosis, cytochalasin D (CytoD), on Day 2 of the treatment (90 ± 3% healthy neurons for sialyl‐transferase inhibitor plus CytoD, and 86 ± 5% healthy neurons for CytoD and sialidase). CytoD alone slightly increased the number of apoptotic neurons, indicating some toxicity, but CytoD also increased the number of live neurons in the presence of sialidase or sialyl‐transferase inhibitor (Figure 4a, gray bars), indicating that desialylation results in microglial phagocytosis of live neurons. In order to test whether this phagocytosis was mediated by CD11b, we added the CD11b‐blocking antibody to the co‐cultures and found that blocking CD11b prevented the loss of neurons induced either by stimulating desialylation or inhibiting sialylation (Figure 4b). A IgG2a isotype control was used to control for any unspecific binding, and had no effect (Figure 4b).

To assess if the observed effects were due to desialylation of microglia rather than desialylation of neurons, we added desialylated primary rat microglia to the glial‐neuronal co‐cultures. Indeed, we found that addition of microglia pretreated with either sialidase or sialyl‐transferase inhibitor induced neuronal loss that could be blocked by co‐treatment with CD11b antibody (Figure 4d). Thus, desialylation of microglia, in the absence of desialylation of neurons, was sufficient to induce neuronal loss mediated by CR3.

### 3.5 LPS‐ or Aβ‐induced neuronal loss in glial‐neuronal co‐cultures, prevented by a sialidase inhibitor DANA or by a CD11b‐blocking antibody

**Figure 5:**
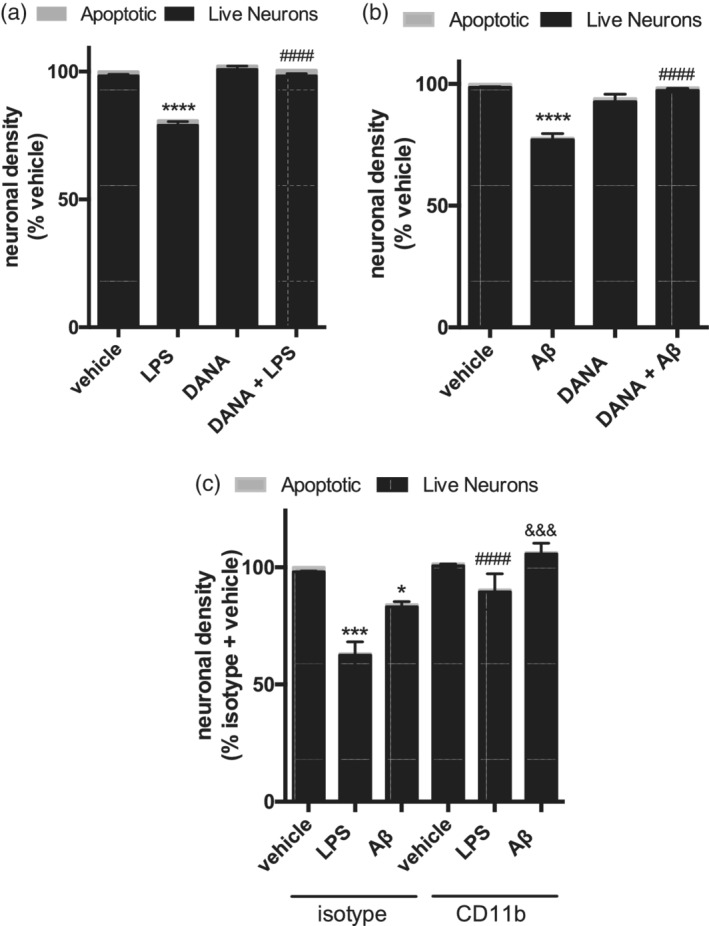
Blockage of sialidase or CD11b prevents LPS‐ or Aβ‐induced neuronal loss. Neuronal densities of mouse‐derived neuronal‐glial co‐cultures that were co‐treated with 100 ng/mL lipopolysaccharide (LPS) (a) or 1 μM fibrillar amyloid beta (Aβ) (b) and pan‐sialidase inhibitor N‐acetyl‐2,3‐dehydro‐2‐deoxyneuraminic acid (DANA, 400 μM) for 3 days. Mean neuronal densities of three independent cultures were quantified and normalized to vehicle condition. Error bars represent *SEM*. Statistical analysis was performed by two‐way ANOVA with Sidak's post hoc test, ****p < .0001, ^####^ *p* < .0001 versus LPS (a) or Aβ (b). (c) Neuronal densities from rat derived neuronal‐glial co‐cultures that were treated for 3 days with LPS (100 ng/mL) or fibrillar Aβ (1 μM). At Day 2 of treatment either isotype control or CD11b‐blocking antibody were added to the cultures and densities assessed at Day 3. Data presented as mean neuronal densities of three independent cultures, normalized to vehicle + isotype control (±*SEM*). Statistical analysis was performed by two‐way ANOVA followed by Sidak's post hoc test, *p < .05, ***p < .001 versus isotype + vehicle, ^####^ *p* < .0001 versus isotype + LPS, ^&&&^ *p* < .001 versus isotype + Aβ

As LPS induced a sialidase activity and desialylation of microglia (Figure 1), and sialidase‐induced desialylation of microglia caused phagocytosis of live neurons (Figure 4), we tested whether LPS could induce neuronal loss via sialidase activity. As expected, addition of LPS or fibrillar Aβ to neuronal‐glial co‐cultures for 3 days resulted in neuronal loss, without increasing neuronal death (Figure 5). Co‐treatment with the sialidase inhibitor DANA at 400 μM completely prevented this loss, again without increasing neuronal death (Figure 5a,b). This indicated that LPS and Aβ induce neuronal loss via an extracellular sialidase activity (as DANA does not enter cells), and thus extracellular sialidases may be treatment targets to prevent inflammatory neuronal loss. Moreover, addition of CD11b‐blocking antibody prevented LPS‐ or Aβ‐induced neuronal loss (Figure 5c), indicating the involvement of CD11b in this inflammatory neuronal loss.

## 4 DISCUSSION

In the fields of macrophage and neutrophil biology, the regulation of functions such as phagocytosis and cell adhesion by cell surface neuraminidase is widely accepted (Sakarya et al., 2004; Seyrantepe et al., 2010). However, this has not previously been investigated in microglia. Previously, we have shown that BV‐2 microglia present a desialylating activity on their cell surface when stimulated with the TLR‐4 agonist LPS (Nomura et al., 2017). In this study, we show that primary microglia activated by LPS, Aβ, TAU or PMA have increased surface sialidase activity and a substantial desialylation of the surface.

In order to model the potential effects of sialidase activity and desialylation of the cell surface, we treated BV‐2 or primary rat microglia with desialylating reagents. Both exogenous sialidase and 3‐FAx‐peracetyl‐Neu5Ac were effective at desialylating microglia, as measured by an increase of PNA binding. Microglial desialylation stimulated microglial phagocytosis of 5 μm carboxylated beads (mimicking the size and charge of cells). A recent study (Pluvinage et al., 2019) found that desialylation of BV‐2 cells stimulated phagocytosis of beads, and this was attributed to Siglec‐2 (sialic acid‐binding immunoglobulin‐type lectin, CD22) inhibiting phagocytosis when microglia are sialylated, but losing this inhibitory activity when microglia are desialylated. Whether CD22 regulates phagocytosis via CR3 is unknown. Siglec‐3 (CD33) has been proposed to have similar inhibitory role to Siglec‐2 (CD22), regulated by sialylation (Bradshaw et al., 2013). The finding that multiple receptors appear to monitor the sialylation state of the microglial surface supports the idea that this sialylation status changes and is important for microglial function.

We found that microglial desialylation increases microglial phagocytosis, and that this increased phagocytosis is mediated by CR3. For example, depletion of CD11b, the alpha‐subunit of the CR3 integrin, blocked sialidase‐induced phagocytosis of beads. And blocking CD11b with a blocking antibody in primary microglia also completely prevented the sialidase‐induced phagocytosis. And microglia treated with sialyl‐transferase inhibitor or sialidase bound more fibrinogen, consistent with activation of CR3. However, we did not test whether the effect of microglial desialylation on CR3 is direct (i.e., due to desialylation of CR3 itself) or indirect (e.g., due to inside‐out activation of CR3). The possibility that desialylation of CR3 itself causes the increased phagocytosis is consistent with literature evidence that activated neutrophils desialylate CD11b and CD18, which apparently activates CR3 binding to ligands (Feng et al., 2011). Thus, there is the possibility that CR3 might be outside‐in activated by sialylation. However, CR3 can be inside‐out activated by multiple signal transduction pathways (Abram & Lowell, 2009) that might be affected by microglial desialylation. Thus, further research is required to determine whether microglial desialylation activates CR3 directly or indirectly.

To test whether desialylation of microglia might induce phagocytosis of live neurons, we induced desialylation of neuronal‐glial co‐cultures. We found a decrease in neuronal density after treatment with either a sialidase or a sialyl‐transferase inhibitor, which was blocked by an inhibitor of phagocytosis cytochalasin D. This indicated that desialylation induced phagocytosis of live neurons. A confounding factor in this experiment is, however, that desialylation of astrocytes and neurons is occurring, as well as microglia. Desialylated neurites are known to be targeted by microglial phagocytosis (Linnnartz et al., 2011; Wielgat & Braszko, 2012). Note, however, that we induced desialylation with an exo‐sialidase, whereas Linnnartz et al., 2011; Wielgat & Braszko, 2012 also added endo‐sialidase to remove 2,8‐linked poly‐sialic acids, which are known to inhibit phagocytosis (Wang & Neumann, 2010). To confirm that desialylation of microglia triggers neuronal loss, we desialylated primary rat microglia using sialidase or sialyl‐transferase inhibitor and added these microglia to neuronal‐glial co‐cultures. We observed a significant reduction in neuronal cell number after 1 day of co‐culture with these desialylated microglia, and this neuronal loss was blocked by co‐treatment with the CD11b antibody. This indicates that CD11b is involved in the removal of neurons from primary cultures by desialylated microglia. However, it is important to note that desialylation (of neurons or microglia) can activate microglial phagocytosis of neurons or neuronal parts (neurophagy) by multiple potential mechanisms, including: (a) increased deposition and activity of complement C1q and C3b (Linnnartz et al., 2011), (b) increased binding of opsonin galectin‐3 (Nomura et al., 2017), (c) decreased trans‐activation of inhibitory Siglec‐11 (Wang & Neumann, 2010), Siglec‐F (Wielgat & Braszko, 2012) and Siglec‐E (Claude, Linnartz‐Gerlach, Kudin, Kunz, & Neumann, 2013), and (d) possibly decreased cis‐activation of inhibitory Siglec‐2 (CD22; Pluvinage et al., 2019) and Siglec‐3 (CD33; Bradshaw et al., 2013).

As LPS‐ or Aβ‐induced microglial desialylation, and desialylation induced neuronal loss, we tested whether LPS‐ or Aβ‐induced neuronal loss via sialidase activity in neuronal‐glial co‐cultures. Both LPS and Aβ are known to induce loss of neurons in these cultures via phagocytosis of live neurons (Neher, Neniskyte, & Brown, 2012; Neniskyte, Neher, & Brown, 2011). Here we show that we can prevent this neuronal loss by co‐treatment with the sialidase inhibitor DANA. Similarly, blockage of CD11b prevents the loss seen when treating with LPS or Aβ. This suggests that LPS‐ and Aβ‐induced neuronal loss is mediated by activation of CD11b by an extracellular sialidase activity. Therefore, inhibition of this sialidase activity is a potential treatment target to prevent inflammation‐induced neuronal loss.

Mutations of sialyl‐transferase genes cause multiple diseases of brain development in humans, and knockout of homologous genes in mice cause loss of myelin, dendrites and neurons (Yoo et al., 2015). This has been suggested to be caused by the desialylation of myelin and neurons, but the results reported here suggest that desialylation of microglia might also contribute to excessive microglial phagocytosis of neurons and potentially myelin and synapses. Synaptic pruning during development is thought be mediated by microglial CR3 (Schafer et al., 2012), and therefore is potentially regulated by microglial desialylation. And excessive synaptic pruning during development may contribute to cognitive impairments and schizophrenia (Sekar et al., 2016), and this is potentially linked to desialylation as educational attainment, cognitive ability, maths ability and/or schizophrenia are associated with genetic variants in sialidases Neu1 and Neu2, and sialyl‐transferases ST3GAL2, ST3GAL3, ST6GAL2, ST8SIA1, ST8SIA2 and ST8SIA4 (https://genetics.opentargets.org). One mechanism by which these enzymes may be regulating brain development and function is by changing the sialylation status of microglia.

## CONFLICT OF INTEREST

The authors declare no competing financial interests.

## Data Availability

The data that support the findings of this study are available from the corresponding author upon reasonable request.
